# Integrated Transcriptomics and Metabolomic Profiling Suggests That Thymol Adaptation Induces Multi-Layered Envelope and Metabolic Perturbations That Sensitize *Pseudomonas psychrophila* to Antibiotics

**DOI:** 10.3390/ijms27093777

**Published:** 2026-04-23

**Authors:** Natacha Caballero Gómez, Wissal Naim, Julia Manetsberger, Carlos Terriente-Palacios, José G. Vallarino, Nabil Benomar, Hikmate Abriouel

**Affiliations:** 1 Area of Microbiology, Department of Health Sciences, Faculty of Health Sciences, University of Jaén, 23071 Jaén, Spain; ncgomez@ujaen.es (N.C.G.); nw000003@red.ujaen.es (W.N.); jmanetsb@ujaen.es (J.M.); nben@ujaen.es (N.B.); 2 Department of Molecular Biology and Biochemistry, Institute of Subtropical and Mediterranean Horticulture ‘La Mayora’, University of Malaga—Consejo Superior de Investigaciones Científicas (IHSM-UMA-CSIC), 29750 Malaga, Spain; cterrient@uma.es (C.T.-P.); vallarino@uma.es (J.G.V.)

**Keywords:** thymol, antibiotics, transcriptomic analysis, *Pseudomonas psychrophila*

## Abstract

The emergence of multidrug-resistant *Pseudomonas* strains poses a serious threat to public health. Essential oil components, such as thymol (TH), exhibit potent antibacterial activity. However, the effects of continuous sublethal TH exposure and resulting changes to antibiotic susceptibility remain poorly understood. Here, we investigated a multi-resistant *Pseudomonas psychrophila* strain after TH adaptation using an integrated transcriptomic and metabolomic approach. Treatment with TH caused a significant decrease in MIC values for aminoglycosides (streptomycin, gentamicin, kanamycin) and tetracycline and increased susceptibility to five other antibiotics. Multi-omics analyses revealed coordinated changes in fatty acid metabolism (*FabI* downregulation and accumulation of unsaturated fatty acids), lipid A biosynthesis (*LpxC* downregulation), peptidoglycan synthesis (*Mur* genes downregulated, accompanied by increased spermine levels), and stress response pathways (such as GABA, GadA, maltose, and MalK). These results suggest that metabolic alterations and envelope remodeling potentially affect cell wall integrity and growth, which could, in turn, contribute to increased antibiotic susceptibility and re-sensitization. Overall, our findings highlight the potential of TH-mediated sensitization as a complementary strategy to restore antibiotic efficacy.

## 1. Introduction

The increasing and accelerated spread of antibiotic-resistant and biofilm-forming bacteria represents a universal threat, with severe potential impacts on the global economy [1]. Therefore, it is essential to find efficient and sustainable strategies to limit antimicrobial resistance (AMR) spread and the increasing occurrence of biofilm-associated infections [2]. Although less well understood than the prominent pathogen *P. aeruginosa*, non-pathogenic Pseudomonas strains have recently also been connected to several human diseases [3]. In particular, multiple antibiotic-resistant *Pseudomonas* spp. strains have been shown to potentially pose serious risks in acute and chronic infections [4]. In addition, the presence of antibiotic resistance genes (ARGs), which can be acquired and transmitted through horizontal gene transfer, further increases the risk associated with *Pseudomonas* spp. This clearly calls for further in-depth studies on the topic [5].

Essential oil components (EOCs), such as thymol (TH), carvacrol, and cinnamaldehyde, have shown promising potential to control multi-resistant *Pseudomonas* spp., both in the planktonic and biofilm state [6,7,8]. In particular, thymol and carvacrol have received great attention due to their antimicrobial effect and beneficial health properties [9,10]. In this context, our previous work [11] has shown that adaptation to TH affects biofilm formation when the strain is subsequently re-exposed to sublethal concentrations of this compound. This could indicate that such adaptation is leading to structural changes related to the cell wall, acting at the level of redox state, energy imbalance, and decreasing the expression of virulence factors. This leads us to hypothesize that the adaptation could affect the antibiotic Minimum Inhibitory Concentration (MICs). In addition, we have previously demonstrated [12] the effects of eugenol adaptation on *Enterococcus* spp. strains leading to changes in antibiotic MICs, as well as a resistance reversion to kanamycin, erythromycin, and tetracycline.

Studies have shown that changes in the specific metabolic spectrum are related to antibiotic resistance [13]. In recent years, more attention has been paid to understanding the relationship between metabolic characteristics and the mechanisms of antibiotic resistance [14,15]. However, only a few studies investigated the underlying mechanisms at the gene expression level. Thus, there is a need to combine and interconnect both techniques (metabolomics and transcriptomics) to analyze drug resistance mechanisms in depth.

Therefore, this study aimed to further our understanding of this field by investigating the effects of TH adaptation on the antibiotic susceptibility profile of *P. psychrophila* M33T02.2. To do so, we compared and correlated transcriptomic and metabolomic changes in the strain. *Pseudomonas psychrophila* M33T02.2 is a multi-resistant and very strong biofilm producer strain [6], which was previously isolated from the white room of a slaughterhouse [16]. Using such a psychrotrophic biofilm-forming strain [17] might have an advantage over other thermoduric microorganisms, as it is able to withstand temperature changes, counteract reactive oxygen species, and endure nutritional starvation [18].

## 2. Results

### 2.1. Phenotypic Response of Pseudomonas psychrophila M33T02.2 to TH Adaptation

The results of antimicrobial susceptibility assays showed that TH adaptation of *P. psychrophila* M33T02.2 decreased the MIC to nine antibiotics, namely, CIP, ciprofloxacin; CL, chloramphenicol; CN, gentamicin; ER, erythromycin; KN, kanamycin; IPM, imipenem; RD, rifampicin; SP, streptomycin; and TE, tetracycline (Table 1). The MIC of KN, TE, CN, and SP decreased by 1200-, 619-, 260-, and 16-fold, respectively, while the MIC of CIP, CL, ER, and IPM decreased in the range of 2–8-fold (Table 1). The phenotype of the TH-adapted *P. psychrophila* strain was reproducibly observed across independent experiments. Following recovery from TH-free glycerol stocks, the MIC values remained consistent at 2500 µg/mL, representing a significant increase from the initial 150 µg/mL MIC of the wild-type strain. It is noteworthy that TH significantly decreased the MIC values for *P. psychrophila* M33T02.2 to CN, KN, SP, and TE. This suggests that exposure of the organism to TH made it more susceptible to these antibiotics. In the other cases (CIP, CL, ER, IPM, and RD), TH-adapted *P. psychrophila* M33T02.2 was more susceptible to the tested antibiotic compared to the non-adapted strain (Table 1).

With regards to the rest of the tested antibiotics—AMX, amoxicillin; AMP, ampicillin; CX, cefuroxime; and F, nitrofurantoin—no changes were evident in their MICs comparing the non-adapted and TH-adapted strains (Table 1). MIC values are reported as quantitative measurements, and no clinical susceptibility categorization was applied, since standardized breakpoints do not exist for *P. psychrophila*.

**Figure 1:**
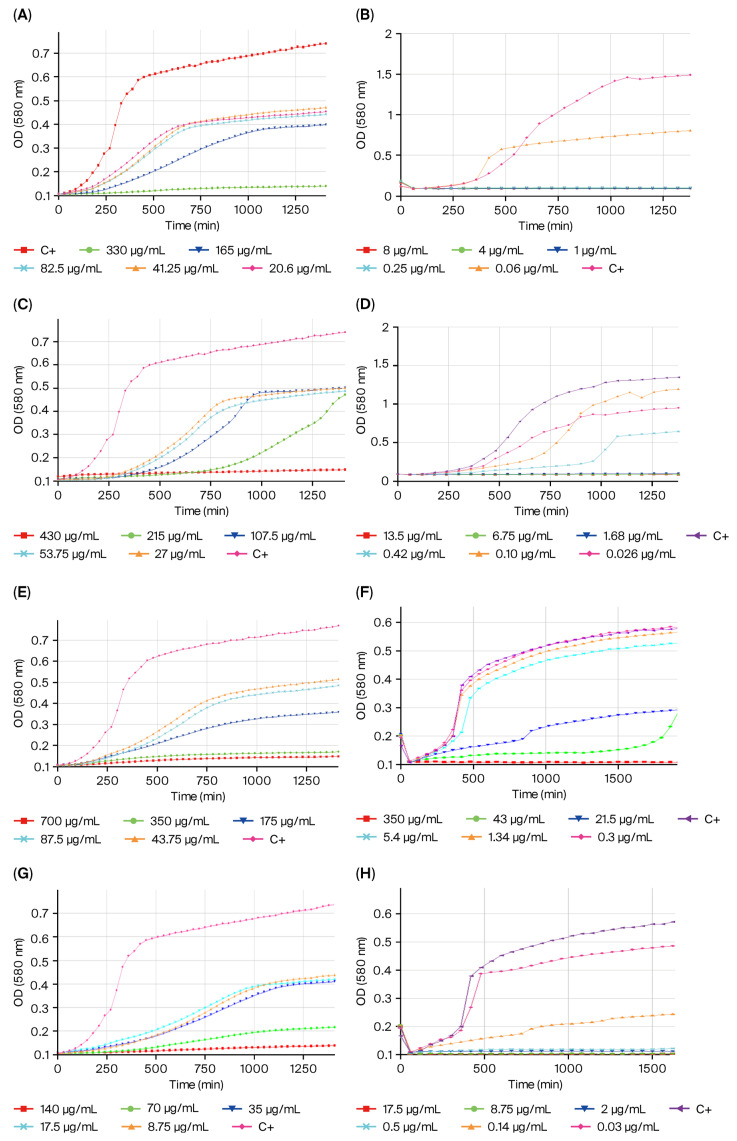
Effect of antibiotics kanamycin (**A**,**B**), tetracycline (**C**,**D**), streptomycin (**E**,**F**), and gentamicin (**G**,**H**) at different concentrations on the growth dynamics of non-adapted (**A**,**C**,**E**,**G**) and TH-adapted (**B**,**D**,**F**,**H**) *Pseudomonas psychrophila* M33T02.2 strains. Each data point (time resolution 72 h:1 h) represents mean values of triplicate cultivations, normalized with data from identical incubations in the absence of bacterial cells (negative controls).

Kinetic studies were used to compare the effect of TH treatment on MIC reductions in a consistent and interpretable manner and confirm MIC reduction (Figure 1). All four antibiotics showed concentration-dependent effects on basal microbial growth dynamics (Figure 1A–H). Furthermore, we observed a short lag phase (4 h) in the absence of the treatment (controls), while it was extended in the presence of antimicrobials. This was particularly obvious in the case of tetracycline exposure (215 µg/mL), which showed the longest lag phase, reaching a duration of 12 h (Figure 1C).

On the other hand, it is interesting to note the different growth pattern of the TH-adapted strain versus the non-adapted strain of *P. psychrophila* M33T02.2. In particular, a longer lag phase is evident in the case of the TH-adapted strain (>4 h for all antibiotics) compared to the wild-type strain (Figure 1).

### 2.2. Monitoring Changes in the TH-Adapted Strain of Pseudomonas psychrophila M33T02.2 Transcriptome

**Figure 2:**
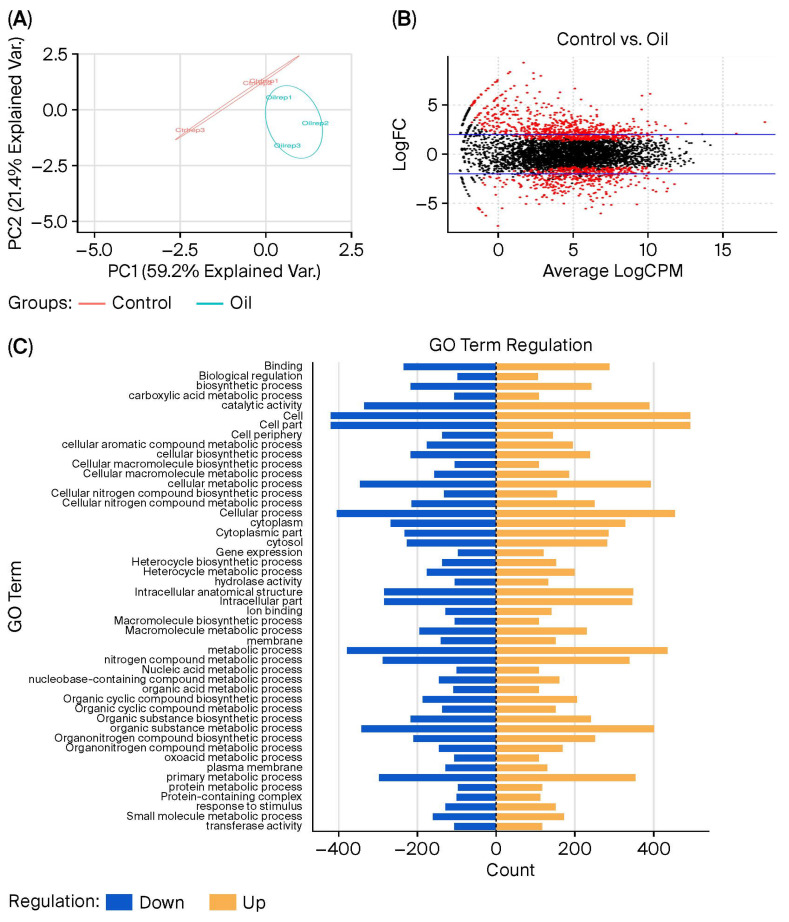
Transcriptomic analysis of non-adapted and TH-adapted *Pseudomonas psychrophila* M33T02.2 strains. (**A**) Principal component analysis (PCA) plot; red plots represent the non-adapted strain and blue plots correspond to the TH-adapted strain. (**B**) MD plot showing the log fold change and average abundance of each gene. Significantly differentially expressed genes (DEGs) are highlighted in red. (**C**) Gene ontology (GO) analysis of significantly differentially expressed genes (DEGs). Significantly upregulated (blue) and downregulated (orange) DEGs (log_2_ fold change > 0.5) between non-adapted and TH-adapted *P. psychrophila* M33T02.2 strains are indicated.

In order to characterize changes in mRNA expression, transcriptomic analysis was performed using the DESeq2 [19] and EdgeR [20] programs, comparing the non-adapted control “C” versus the “TH-adapted” conditions. Principal component analysis yielded two components: the first principal component (PC1) explained 59.2% of the variability between samples, and the second principal component (PC2) explained 4%. Two separate experimental groups (non-adapted “C” and TH-adapted “Oil”) could be identified in a clear-cut way (Figure 2A). As shown in Table 2 and Table S1, 1721 differentially expressed genes were common to both methods, with 682 genes downregulated and 1039 upregulated.

Gene ontology (GO) analysis (Figure 2C) showed that the TH-adapted strain was mainly affected positively (K) by transcription, followed by (E) amino acid metabolism and transport and (P) inorganic ion transport and metabolism. These pathways were the most upregulated metabolic processes represented by 103, 98, and 94 overexpressed genes, respectively. Next, (M) cell wall/membrane/envelope biogenesis, (J) translation, ribosomal structure and biogenesis, and (T) signal transduction mechanisms were also associated with overexpression of 74, 67, and 56 genes, respectively (Table S1, Supplementary Materials). On the other hand, downregulation of genes followed the same dynamic, with (K) transcription, (P) inorganic ion transport and metabolism, and (E) amino acid metabolism and transport as the most prominent down-regulated functions related to 71, 65, and 62 down-expressed genes (Table S1, Supplementary Materials). However, focusing on the balance between overexpressed and downregulated genes related to (L) replication, recombination, and repair, (I) lipid transport and metabolism, and (Q) secondary metabolites biosynthesis, transport, and catabolism pathways, we could observe that the TH-adapted strain showed an equilibrium between the number of expressed genes (Table S1, Supplementary Materials). Generally, as visible from Figure 2C, the main biological pathways upregulated in the TH-adapted strain were transport pathways and transcription metabolism. However, it is remarkable that the pathway associated with cell division and the cell cycle was clearly downregulated (Table S1, Supplementary Materials).

KEGG pathway annotation results identified 282 down-regulated and 433 up-regulated differentially expressed genes in bacterial metabolism, accounting for 41% of the total. There were 40 differentially expressed genes related to membrane transport function (Table S1, Supplementary Materials).

With respect to putative ARGs, eight genes were differentially expressed in the TH-adapted strain. Hence, these were selected for further analysis. Among the four downregulated genes, bioinformatic annotation suggested potential associations with resistance to aminoglycosides (*creatinase* gene), beta-lactam (*fliM* gene), and multidrug (*acoR_2* and *sfnC_5*) resistance. These putative multidrug resistance functions are predicted to encompass a broad range of agents, including dyes, tetracycline, fluoroquinolones, peptides, and macrolides, among others (Table 3). Conversely, four upregulated genes with putative roles in resistance to multidrug (*pse_5134* gene), peptide (*dapL* gene), beta-lactam (*yecD* gene), and other agents (*dctD_4* gene) were also identified (Table 3). It should be noted that these functional assignments represent bioinformatic predictions and require further experimental validation to confirm their specific contribution to the observed phenotype.

### 2.3. Metabolomic Analysis

**Figure 3:**
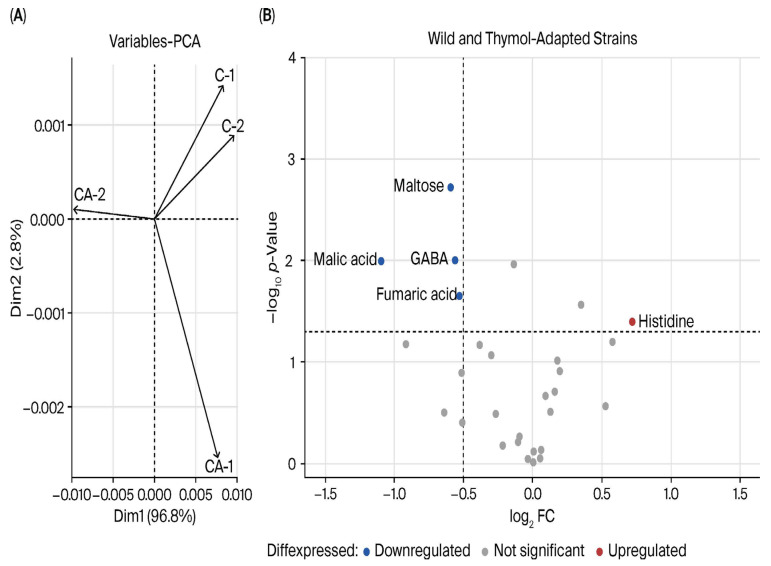
Metabolomic analysis of non-adapted and TH-adapted *Pseudomonas psychrophila* M33T02.2 strains using GC-ToF-MS and UHPLC-Q-Orbitrap-MS/MS. (**A**) Principal component analysis (PCA) plot using GC-ToF-MS. (**B**) Volcano plot of metabolites, the abscissa is the FC value of log_2_, and the ordinate is the *p*-value of −log_10_, which makes the gap between the substances with great differences large and the substances with small differences narrow.

**GC-ToF-MS analysis**. Metabolomic studies revealed differences between the non-adapted and the TH-adapted *P. psychrophila* M33T02.2 strains. The metabolite profiles of both strains were determined, with 44 metabolites identified, 14 of them using a putative annotation (Table S2, Supplementary Materials). A principal component analysis (PCA) was carried out for all metabolites. As a result, when comparing *P. psychrophila* M33T02.2 wild-type and TH-adapted strains (Figure 3A), the PCA score was 96.8%, and the samples in each group were clustered. This analysis showed clear differences between control and TH-adapted strains.

Student’s *t*-test analysis was performed to detect differences between non-adapted and TH-adapted *P. psychrophila* M33T02.2 groups and fold change value (log_2_ FC value) (Table 4 and Table S2). This comparison revealed differences for malic acid, histidine, maltose, gamma-aminobutyric acid (GABA), and fumaric acid (Table 4). As shown in Figure 3B, malic acid, maltose, GABA, and fumaric acid were downregulated in TH-adapted *P. psychrophila* M33T02.2, and only histidine was upregulated.

**Figure 4:**
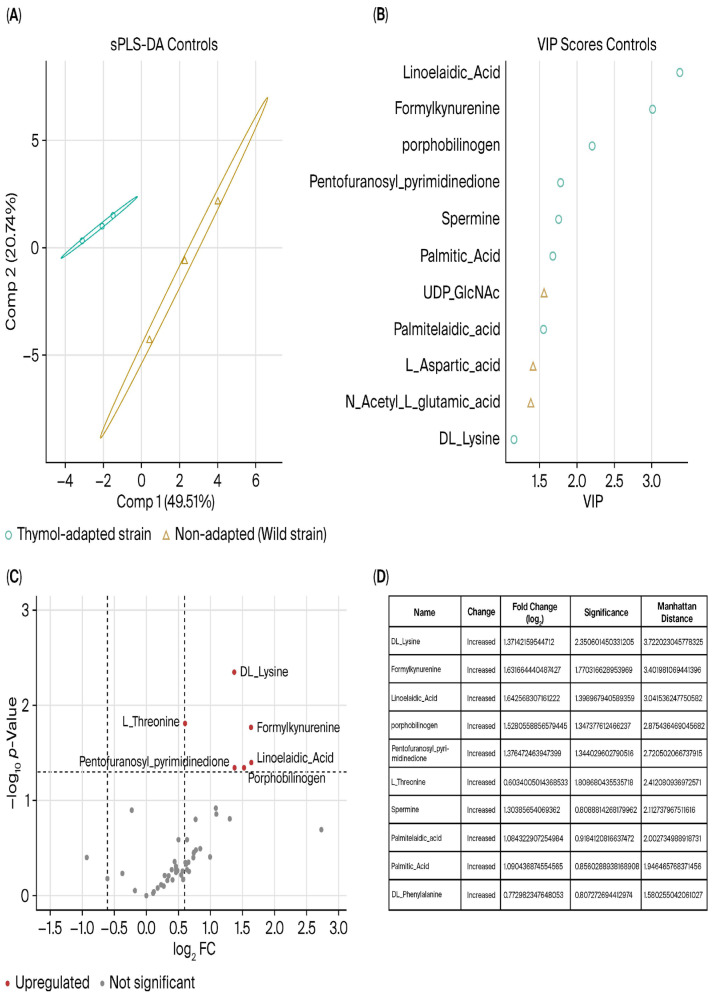
UHPLC-Q-Orbitrap-MS/MS analysis. (**A**) PLS-DA plot; the blue dots represent TH-adapted *P. psychrophila* M33T02.2 strain, and the orange triangles represent the non-adapted *P. psychrophila* M33T02.2 strain. (**B**) Variable importance in projection (VIP) coefficient. (**C**) Volcano plot of metabolites, the abscissa is the FC value of log_2_, and the ordinate is the *p*-value of −log_10_, which makes the gap between the substances with great differences large and the substances with small differences narrow. (**D**) Summary for the plotted increase in metabolites and the statistical information.

**UHPLC-Q-Orbitrap-MS/MS analysis**. In order to perform a more in-depth metabolite analysis, UHPLC-Q-Orbitrap-MS/MS was used. A total of 51 polar and semi-polar metabolites were annotated as putative (Table S3, Supplementary Materials). To better understand the significance of these metabolites in the TH-adapted strains’ metabolic response, sparse partial least squares (sPLS) and sparse partial least squares discriminant analysis (sPLS-DA) were performed. The sPLS-DA components were obtained and plotted (Figure 4A), and the importance of the different metabolite matrices was demonstrated. There was a clear separation between the two groups, indicating metabolic differences between the wild-type and the TH-adapted *P. psychrophila* M33T02.2 strains. Graphical PLS-DA loading of metabolites in each component of the model was also obtained (Figure S1, Supplementary Materials). Furthermore, in order to identify significantly different metabolites between the two groups, the variable importance in projection (VIP) coefficient was obtained for each metabolite, with those having values over 1 being represented (Figure 4B). These metabolites were mainly related to long-chain fatty acids, amines, and amino acids (DL-Lysine, L-Aspartic acid, and N_Acetyl_L_glutamic_Acid) metabolism.

**Figure 5:**
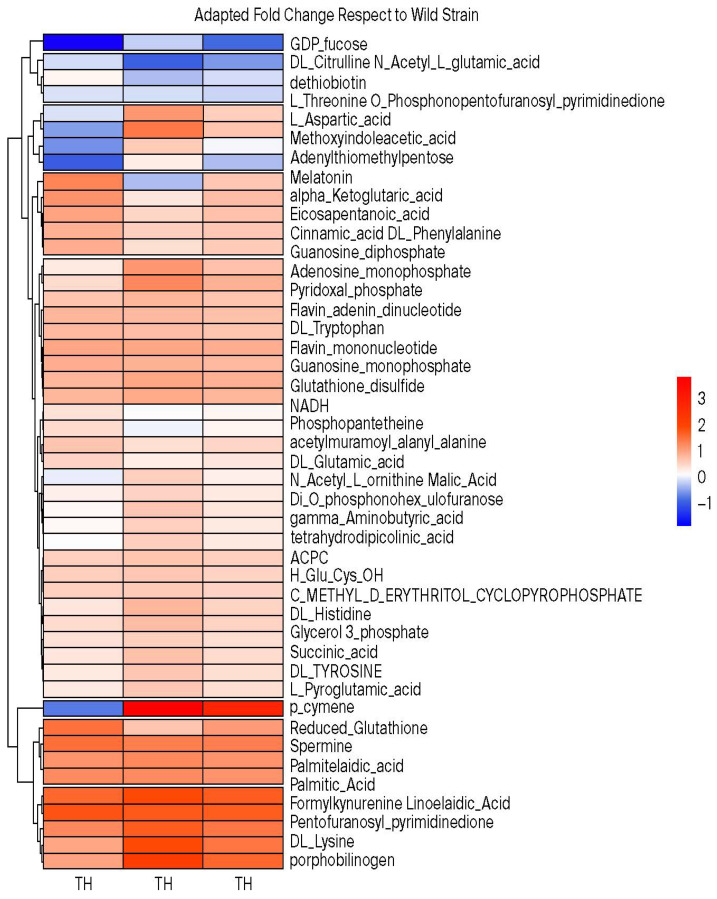
Hierarchical cluster analysis (HCA) and heatmap visualization of changes in the relative abundance of annotated semi-polar metabolites obtained by UHPLC-Q-Orbitrap MS/MS analysis. Each cell represents the log_2_ of the fold change (FC), with red and blue colors denoting relatively high and low intensities, respectively. Metabolites are grouped by clusters, using Pearson correlation coefficients.

Metabolic differences between non-adapted and TH-adapted *P. psychrophila* M33T02.2 strains were confirmed using one-step analysis applied to screen the marker differential metabolites. Student’s *t*-test was used to identify the differences between the two groups. The calculation of the metabolite abundance ratio between the two groups, the difference multiple, or fold change value (FC value) was obtained. By combining the *p*-value and FC value for each metabolite, a volcano map of metabolites was drawn (Figure 4C,D). The results showed that there are significant differences in metabolites between the non-adapted and the TH-adapted strain. Ten metabolites were found at significantly higher levels in the TH-adapted strain (FC value greater than 0.5), accounting for 20% of the total identified metabolites. These included linoelaidic, palmitic, and palmitelaidic acids, as well as spermine and various amino acids (fold change values are presented in Figure 4 and their abundance profiles in Figure 5).

Metabolites were interpreted according to MSI annotation levels, with level 2 considered putatively identified compounds and level 3 treated as putative annotations. Model performance for sPLS-DA was validated using cross-validation, with classification error rate and Q^2^ values calculated. Permutation testing further confirmed model robustness, supporting the reliability of the identified differential metabolites.

In summary, 51 metabolites were selected for the implementation of hierarchical Pearson clustering (Figure 5), revealing a statistically significant discrepancy between the wild-type and the TH-adapted *P. psychrophila* M33T02.2 strains. This finding was consistent with the outcomes of the PLS-DA analysis. Long-chain fatty acids, palmitic, palmitelaidic, and linoelaidic, as well as spermine, stood out as overproduced metabolites with respect to the control with fold changes over 1 (Figure 5), corresponding with VIP values over 1 in the PLS-DA model.

## 3. Discussion

Due to the spread of “superbugs” at an alarming rate, new therapeutic approaches are urgently needed [21]. Efficient indirect antibacterial strategies could provide a solution to maintain the therapeutic efficiency of existing antibiotics [22]. Our previous study [12] showed that Eugenol could be used in disinfectant formulations for reversing antibiotic resistance. TH adaptation has been previously shown to remodel the physiology and metabolic profile of *P. psychrophila* M33T02.2, leading to reduced biofilm formation and altered cellular behavior [11]. Therefore, we considered it crucial to explore the mechanisms involved in the development of adaptation to TH. These findings, combined with the growing interest in essential oil compounds, like TH, for their potential activity against pathogenic bacteria, led us to conduct a multi-omic study on repeated exposure to TH to reveal its role in increasing antibiotic susceptibility.

The phenotype of the TH-adapted strain was consistently observed following recovery from thymol-free glycerol stocks, suggesting that the effects were not caused by residual thymol. In this context, the term “adaptation” is used to describe a reproducible phenotypic state observed under the experimental conditions tested and does not imply confirmed genetic fixation. While the term “adaptation” is employed throughout the manuscript, it specifically refers to this persistent phenotypic state, as formal long-term stability or genetic fixation studies were not conducted. Susceptibility testing revealed that TH-adaption of *P. psychrophila* M33T02.2 led to substantial MIC reductions. Specifically, increased susceptibility was observed for four of the thirteen antibiotics tested, particularly aminoglycosides (streptomycin, gentamicin, and kanamycin) and tetracycline. Additionally, five other agents (CIP, CL, ER, IPM, and RD) showed a 2- to 8-fold increase in susceptibility. The highest susceptibility increases were confirmed by bacterial growth kinetic studies, which indicated extensions in the lag phase of the TH-adapted strain, possibly related to metabolic changes. Aduru et al. [23], using modeling studies, demonstrated that under pressure selection at sub-inhibitory drug concentrations, antibiotic-adapted cells were able to grow more efficiently, highlighting that the metabolism can act as a selective target independent of both growth rate and the primary antibiotic mechanism. However, the TH-adapted *P. psychrophila* M33T02.2 showed lower growth efficiency, indicating that TH adaptation does not follow the same behavior as adaptation to antibiotics. In order to elucidate the main metabolic pathways involved in antibiotic susceptibility caused by TH-adapted *P. psychrophila* M33T02.2, a combined transcriptomics and metabolomics approach was developed.

Firstly, transcriptomic analysis showed that the increase in antibiotic susceptibility was associated with the decreased differential expression of specific genes targeting resistance to aminoglycosides, tetracycline, and other drugs. Among these, *creatinase*, *acoR_2*, and *sfnC_5* genes were identified and may explain the decreased MICs to the tested antibiotics (SP, TE, KN, CN, CIP, CL, ER, IPM, and RD). Furthermore, *acoR_2* and *sfnC_5* genes are also involved in virulence, and thus TH adaptation could allow the decrease in *Pseudomonas* pathogenesis. Audrain et al. [24] reported the impact of bacterial volatile compounds (such as acetoin) on bacterial biology through modulation of antibiotic resistance, biofilm formation, and virulence. Under TH treatment, *acoR_2* was downregulated, whereas *acoB* exhibited a 1,7-fold upregulation (log_2_ FC). Although these genes are annotated within the same operon, RNA-seq analysis provides gene-level quantification. Differential expression within operons is a well-documented phenomenon often attributed to transcript processing or stability differences rather than independent regulation. Since *acoR_2* was the only determinant in this cluster identified in our bioinformatic pipeline as a regulatory factor associated with pathogenicity/ARG-related categories, our discussion focuses on its specific modulation rather than the global repression of acetoin catabolism. On the other hand, the *sfnC_5* gene, coding for a putative FMNH2-dependent monooxygenase *SfnC*, was reported as an initiator in sulfonamide catabolism, in addition to other metabolic pathways, such as those associated with virulence.

In addition to examining specific antibiotic resistance genes (ARGs), other mechanisms are likely involved. Therefore, metabolomic analysis was necessary for a more complete understanding and to discover new antibacterial targets. The PLS-DA results derived from UHPLC-Orbitrap-MS/MS metabolites yielded three long-chain fatty acids as VIPs in TH-adapted *P. psychrophila* M33T02.2: linoelaidic, palmitic, and palmitelaidic acid. Bacterial fatty acid synthesis is essential for many pathogens, and its synthesis is a desirable target for antibiotic discovery at this moment [25,26]. Kenny et al. [27] showed that linoleic acid can alter the genes involved in peptidoglycan (PG) synthesis in *Staphylococcus aureus*. On the other hand, the increase in PG precursors such as pentaglycine, lysine, glutamate, D-alanine, L-alanine, and others in the presence of linoleic acid could represent a response of the cell to PG inhibition [28]. In this context, DL-Lysine, L-Aspartic acid, and N_Acetyl_L_glutamic_Acid are present among the 11 metabolites determined as VIPs in our study. Furthermore, the transcriptomic results confirmed downregulation of *FabI* (−4.2, log_2_ FC) essential for growth, which implies an inhibition of PG synthesis, as confirmed by Casillas-Vargas et al. [27]. In another study, Zheng et al. [29] reported that linoleic acid inhibited bacterial enoyl-acyl carrier protein reductase (*FabI*). Interestingly, *FabI* inhibitors are currently being evaluated in clinical trials [30,31]. Consistent with the downregulation of *FabI*, our metabolomic analysis revealed an increased abundance of unsaturated fatty acids, suggesting a shift in membrane lipid composition. In parallel, transcriptomic data showed underexpression of *lpxC* (−2log_2_ FC), a key enzyme in lipid A biosynthesis. Because *FabI* catalyzes a committed step in saturated fatty acid biosynthesis and *LpxC* catalyzes the first committed step in lipid A synthesis, the combined effects likely reduce the availability of saturated fatty acids for lipid A assembly and decrease lipid A disaccharide levels [32,33]. Such alterations in lipid composition and LPS structure may compromise outer membrane integrity, enhancing membrane permeability and potentially facilitating antibiotic uptake. These results could help shed light on the mechanism behind the increased susceptibility to antibiotics in the TH-adapted strain.

Spermine was a key metabolite discriminating between wild-type and TH-adapted strains. In Gram-negative bacteria, spermine and putrescine contribute to cell surface integrity and normal growth [34,35]. In the TH-adapted strain, spermine levels increased, likely representing a compensatory response to TH-adapted envelope stress rather than enhanced protection.

Polyamines stabilize the outer membrane by binding LPS and modulate antibiotic susceptibility [36,37]. Our transcriptomic data showed downregulation of the *PotABCD* polyamine transporter (*potA1*, *potA2*, *potA4*) and five *Mur* genes involved in peptidoglycan biosynthesis, which are known to be co-regulated. The concomitant repression of polyamine transport and peptidoglycan synthesis suggests that TH disrupts envelope homeostasis, facilitating the increased antibiotic susceptibility. Given that *Mur ligases* are established antibacterial targets, TH may functionally mimic *Mur* pathway inhibition via polyamine regulation.

In addition to envelope remodeling and polyamine regulation, TH adaptation also impacted central metabolic pathways. Using a complementary metabolomic approach based on GC-ToF-MS, γ-aminobutyric acid (GABA) was identified as a differentially expressed metabolite, showing a decrease of 0.559 log_2_ fold change. This finding was consistent with UHPLC-Q-Orbitrap-MS/MS data, where GABA contributed to group separation in components 2 and 3 of the sPLS-DA model. GABA is involved in nitrogen and carbon metabolism in microorganisms and is linked to polyamine metabolism, including putrescine and spermine. Its intracellular concentration varies under environmental stress and reflects metabolic activity and adaptability. Moreover, GABA participates in the glutamine/glutamate-dependent acid resistance system (AR2) in *Escherichia coli* and other bacteria [38]. Consistently, our transcriptomic analysis revealed downregulation of the glutamate decarboxylase gene *gadA* (log_2_ FC −1.262), which catalyzes the irreversible decarboxylation of L-glutamate to GABA, indicating suppression of the acid resistance response in the TH-adapted strain. This suggests that TH broadly compromises both envelope integrity and central metabolic stress responses, further contributing to growth impairment and increased antibiotic susceptibility.

On the other hand, overexpression of histidine was detected in the TH-adapted *P. psychrophila* M33T02.2 strain. In this context, Zhang et al. [39] demonstrated that D-histidine downregulated the mRNA expression of virulence and quorum-sensing (QS)-associated genes in *P. aeruginosa* PAO1 without affecting bacterial growth. Hence, we can consider this metabolite as an antibiotic potentiator modulating bacterial membrane stability and permeability, ultimately increasing antibiotic susceptibility. This is in agreement with our previous data of TH-adapted strain re-exposed to sublethal concentration, exhibiting reduced biofilm biomass, irregular biofilm architecture, and decreased production of rhamnolipids, which are essential for biofilm maturation and swarming motility [11]. In this sense, the transcriptomic profile (downregulation of *fliM*, *fliE_2*, *fliG_2*, and *flgB* genes) provides a mechanistic explanation for these changes, showing a clear shift away from a motile/biofilm-forming lifestyle, in line with our previous findings [11]. Collectively, these multi-layered disruptions in envelope integrity, polyamine content, nucleotide metabolism, and fatty acid composition provide a plausible mechanistic explanation for both the defective biofilm phenotype and the observed increase in antibiotic susceptibility.

## 4. Materials and Methods

**Bacterial strain and culture conditions**. *Pseudomonas psychrophila* M33T02.2 [16] was used in this study under standard and adapted conditions. Thymol (TH, essential oil component “EOC”) adaptation of *P. psychrophila* M33T02.2 was achieved by inoculating 2 mL of an overnight culture (0.5 McFarland turbidity units) with sub-inhibitory concentrations of TH (Sigma-Aldrich, Madrid, Spain) at ½ MIC (“Minimum Inhibitory Concentration”) in Tryptone Soy Broth (TSB; Scharlab, Barcelona, Spain). The bacterial culture was then allowed to grow for 24–48 h at 25 °C. This inoculation was repeated six times in fresh TSB broth [40], where the concentration of TH was progressively increased to a final concentration of 2500 μg/mL (TH-adapted strain). The TH MIC was previously determined by Caballero Gómez et al. [6]. Both TH-adapted and wild-type (non-adapted) strains were stored at −80 °C in TSB supplemented with 20% glycerol in the absence of TH. Strains were recovered in TH-free broth prior to all experiments to eliminate potential TH carryover effects. In this study, the term “adaptation” refers to a reproducible phenotypic state achieved after TH exposure that remains consistent after recovery in TH-free media. Strains were routinely cultured in TSB medium at 25 °C for 24 h if not stated otherwise.

The selection of *P. psychrophila* M33T02.2 strain and the EOC was based on promising results obtained previously regarding biofilm inhibition and the synergistic effects with different antimicrobials [6].

**Antibiotics**. The following thirteen antibiotics obtained from Sigma-Aldrich (Madrid, Spain) and belonging to different classes were tested: ampicillin (AMP), amoxicillin (AMX), gentamicin (CN), ciprofloxacin (CIP), tetracycline (TE), rifampicin (RD), erythromycin (ER), nitrofurantoin (F), chloramphenicol (CL), imipenem (IPM), cefuroxime (CX), kanamycin (KN), and streptomycin (SP). The antibiotic stock solutions were prepared according to the guidelines of the Clinical and Laboratory Standards Institute (CLSI).

### 4.1. Monitoring of the Effect of TH Adaptation on Pseudomonas psychrophila M33T02.2 Antibiotic Susceptibility

**Antimicrobial susceptibility assays**. CLSI M07-2A standard methods were used to determine the antibiotic MICs in TH-adapted and non-adapted *P. psychrophila* strains [41]. 2-fold serial dilutions of compounds at concentrations ranging from 2000 to 0.01 µg/mL were tested. Bacterial cultures grown at 25 °C for 24 h were diluted 1/10 (*v*/*v*) in fresh Mueller–Hinton II (MHII) broth (0.5 McFarland). 20 µL of bacterial culture were distributed in a 96-well microtiter plate (TPP, Trasadingen, Switzerland), before adding 180 µL of MHII broth supplemented with different concentrations of antibiotics. Plates were incubated for 24 h under aerobic conditions. MIC determinations were performed using three independent biological replicates. A positive control (bacteria, no antibiotic) and a negative control (media only) were used. Reproducibility and quality control strain *P. putida* CECT 324 was used.

**Kinetic studies**. Bacterial growth was monitored at 25 °C ± 0.3 °C for 24 h in microtiter plates, measuring the optical density (OD) at 580 nm on a Tecan Infinite M200 multimode microplate reader equipped with monochromator optics (Tecan Group Ltd., Männedorf, Switzerland). Orbital shaking conditions were applied before each measurement (4 mm amplitude and 15 s shaking cycles). Measurements were done once per hour using the multiple reads per-well mode (filled-circle alignment, 3 × 3 spots, five reads per well, border 2000 mm). Each experiment was performed in triplicate.

### 4.2. Omic Studies of the TH-Adapted Pseudomonas psychrophila M33T02.2 Strain Versus the Non-Adapted Strain

**Transcriptome analysis**. Total RNA was isolated from three biological replicates of TH-adapted and non-adapted *P. psychrophila* M33T02.2 using RNase Micro Kit (QIAGEN, Hilden, Germany) according to the manufacturer’s instructions and as described by Alonso García et al. [42] with some modifications. Briefly, RNA was extracted by addition of RNA protect bacteria reagent (QIAGEN, Hilden, Germany), 500 μL of TESL (25 mM Tris, 10 mM EDTA, 20% sucrose, and 20 mg/mL lysozyme; all from Sigma-Aldrich (St. Louis, MO, USA)), and 20 μL mutanolysin (20 U) to cell pellets (from 2 mL overnight culture), followed by incubation at 37 °C for 60 min with gentle shaking. DNaseI digestion was performed according to the manufacturer’s instructions Panreac AppliChem (Barcelona, Spain). RNA quantification and quality assessment were achieved using a NanoDrop 2000 spectrophotometer (Thermo Scientific (Waltham, MA, USA)), and samples were frozen at −80 °C until required.

Six libraries were generated from a total of 50–500 ng, using the Illumina Stranded Total RNA Prep (Illumina, San Diego, CA, USA) and ligation with Ribo-Zero Plus Microbiome ((Illumina, San Diego, CA, USA). The RNA library preparation, sequencing, and bioinformatic analysis were performed at ADM Biopolis S.L. (Valencia, Spain), as described by Alonso García et al. [42]. Raw reads were quality filtered with PRINSEQ http://prinseq.sourceforge.net/ (accessed on 3 June 2025) (minimum read length ≥50 bp; reads with >15% Ns removed). A mixed approach was employed in this study. Initially, taxonomic identification of the samples was performed using the 16S rRNA gene, which indicated that the taxa most closely related to our strain are *Pseudomonas psychrophila* KM02 (GCF_011040435.1) and *Pseudomonas helleri* NF1a (GCF_045830615.1). Subsequently, mapping was conducted using Bowtie2 [43], aligning all samples against the reference transcriptomes of both species. After resolving the mapping and analyzing the resulting mapping files, we identified genes whose presence is clearly supported, with a sequence length of at least 70% relative to the reference and a sequence identity of at least 70%. Following this, a second round of mapping was performed, this time exclusively against the sequences identified as present. From this mapping, unmapped reads were extracted, and these reads were assembled de novo using the RNAseq function of the SPAdes (https://ablab.github.io/spades/ accessed on 3 June 2025) [44] suite. The identified transcripts were filtered based on a minimum length of 60 bp and a minimum coverage of 5, meaning that at least five reads supported the existence of the transcript. The identified genes were then annotated using EggNOG (http://eggnog.embl.de/ accessed on 3 June 2025) (COG/GO/KEGG/PFAM/CAZy) [45] and PathoFact (https://pathofact.lcsb.uni.lu accessed on 3 June 2025) (virulence, toxins, AMR, MGE) [46].

With respect to bioinformatic analysis, two approaches were employed for the expression quantification. The approach involved mapping and quantification utilizing transcript alignment and quantification with Salmon (quasi-mapping) [47] against the mixed reference transcriptome described above. The differential expression tests were performed using the DESeq2 [19] and EdgeR [20] programs, comparing “Control vs. TH” conditions. These analyses examined the differential expression patterns of the identified sequences, either with the Salmon or Corset count data. To ensure high confidence in the reported DEGs, a consensus-based filtering step was applied; only genes consistently identified across both independent bioinformatic pipelines (defined as “COMMON” genes) were selected for further biological discussion. The analyses were performed using Stringtie [48], Ballgown [49], DESeq2, and EdgeR [20]. In this sense, differential expression testing for the primary pipeline was carried out in DESeq2, comparing “TH vs. Control.” Unless otherwise specified, genes were considered differentially expressed (DEGs) when they met both criteria:

- |log_2_ fold change| > 0.5;
- FDR-adjusted *p*-value (Benjamini–Hochberg) < 0.05.

Regarding gene ontology, two analyses [50] were performed. Firstly, for all comparisons made, an analysis based on all genes/transcripts tested was conducted using the R package Goseq (https://bioconductor.org/packages/release/bioc/html/goseq.html accessed on 3 June 2025) [51] to determine differential enrichment of GO categories. Subsequently, a functional analysis of the differentially expressed transcripts was performed to determine the distribution of functional categories in each GO ontology (Cellular Component, Biological Process, and Molecular Function).

Metabolic analysis based on all genes/transcripts tested was conducted using the R package Goseq [51] to determine metabolic enrichment of the differentially expressed genes/transcripts. Information was retrieved from the different metabolic pathways through the download of KEGG maps [52] using the enzyme codes (ECs) associated with each gene/transcript. Additionally, an annotation of these pathways was performed using the GOSEQ program, based on the assignment of metabolic pathways assigned to each gene/transcript.

To avoid favoring lowly expressed genes and also failing to control the false discovery rate (FDR) correctly, a more rigorous approach was applied, modifying the statistical test so as to detect expression changes greater than a specified threshold. These analyses examined the differential expression of mRNA and transcripts. (log_2_ fold change) > 0.5 and *p*-value < 0.05 were considered as differentially expressed of the identified sequences, utilizing both the Salmon and Corset count data. Finally, the results were compared to identify genes that were consistently recognized as differentially expressed by both methods.

The raw data for this study have been deposited in the European Nucleotide Archive (ENA) at EMBL-EBI under accession number PRJEB86510.

**Metabolomic studies of the TH-adapted *Pseudomonas psychrophila* M33T02.2 strain versus the non-adapted strain**. To study the metabolic effects of TH adaptation on *P. psychrophila* M33T02.2, metabolite profiling was performed to identify and annotate primary metabolites by GC-ToF-MS and to annotate polar and semi-polar secondary metabolites by UHPLC-Q-Orbitrap-MS/MS (Supplementary Material). The wild-type and TH-adapted strains were analyzed after incubation at 25 °C for 24 h and subsequent 1/10 (*v*/*v*) dilution in 15 mL of TSB, corresponding to an inoculum density of 0.5 McFarland. Three replicates of each sample (condition) were centrifuged for 15 min at 15,000 rpm, and pellets were stored at −80 °C until analysis.

**Statistical analysis**. Statistical analyses were performed using Excel 2016 (Microsoft Corporation, Redmond, WA, USA) to determine averages and standard deviations in growth kinetic studies with three independent biological replicates. All analyses were performed in triplicate.

## 5. Conclusions

The integration of metabolomic and transcriptomic data indicates that TH exerts multi-layered effects on *P. psychrophila* M33T02.2. Downregulation of *fabI* and *lpxC*, together with increased unsaturated fatty acids, suggests perturbation of lipid A biosynthesis and outer membrane destabilization. Concurrent increases in spermine levels and downregulation of *Mur* genes compromise peptidoglycan synthesis, while decreased GABA and *gadA* expression reflect impaired acid stress response and nitrogen metabolism. Altered maltose metabolism, including differential maltose abundance and *malK* upregulation, highlights a shift in carbohydrate utilization. Collectively, these coordinated alterations in envelope structure, polyamine levels, and central metabolism likely drive increased membrane permeability and heightened susceptibility to multiple antibiotics, offering a mechanistic framework for the observed TH-mediated effects.

## Figures and Tables

**Table 1:** The effect of TH adaptation on the antibiotic susceptibility of *Pseudomonas psychrophila* M33T02.2 isolated from a local goat and lamb slaughterhouse in Jaen (Spain). Data represent mean values from three independent biological replicates.

*Pseudomonas* Strains		MIC of Antibiotics * (µg/mL)												
		AMP	AMX	CIP	CL	CN	CX	ER	F	IPM	KN	RD	SP	TE
***P. psychrophila* M33T02.2**	**Wild strain**	640	350	0.2	700	**130**	1800	125	3	0.4	**300**	4.8	**350**	**260**
	**TH-adapted**	640	350	0.0312	157	**0.5**	1800	31.25	3	0.2	**0.25**	0.6	**21.5**	**0.42**
***P. putida* CECT 324**		250	125	0.2	62.5	15.6	1800	125	3	0.4	1.9	1.9	62.5	31.2

*: AMX, amoxicillin; AMP, ampicillin; CIP, ciprofloxacin; CL, chloramphenicol, CN, gentamicin; CX, cefuroxime; ER, erythromycin; F, nitrofurantoin; IPM, imipenem; KN, kanamycin; RD, rifampicin; SP, streptomycin; TE, tetracycline. The bold indicate antibiotics for which kinetic studies were used.

**Table 2:** Number of differentially expressed genes per comparison.

Quantification Method	DiffExp.Method	All	Negative *	Positive **
**Salmon**	DESeq2	5018	1712	2022
	EdgeR	5161	1769	2081
	Common	1721	682	1039

* Genes/transcripts least expressed (with log_2_ fold change below −0.5 and *p*-value lower than 0.05) under TH adaptation (**negative**). ** Genes/transcripts most expressed (with log_2_ fold change over 0.5 and *p*-value lower than 0.05) under TH adaptation (**positive**).

**Table 3:** Antibiotic resistance genes (ARGs) differentially expressed in TH-adapted *Pseudomonas psychrophila* M33T02.2.

Gene Symbol/Name	Description	log_2_ FC	COG Category	ARG	AMR Category	AMR Subclass	Predicted Associated Mechanism
**Downregulated ARGs**							
***fliM***	Flagellar motor switch protein FliM	−3.076	N	OMPR	Beta-lactam	Carbapenem; penam; cephalosporin; penem; cephamycin; monobactam	Reduced permeability to antibiotics
***acoR_2***	Acetoin dehydrogenase operon transcriptional activator AcoR	−1.798	KQ	ADEF	Multidrug	Fluoroquinolone antibiotic; tetracycline antibiotic	Antibiotic efflux
***creatinase***	-	−2.228	E	KASUGAMYCIN_RESISTANCE_PROTEIN_KSGA	Aminoglycoside	Aminoglycoside antibiotic	-
***sfnC_5***	putative FMNH2-dependent monooxygenase SfnC	−1.004	I	OPRM	Multidrug	Acridine dye; aminocoumarin antibiotic; aminoglycoside antibiotic; carbapenem; cephalosporin; cephamycin; diaminopyrimidine antibiotic; fluoroquinolone antibiotic; macrolide antibiotic; monobactam; penam; penem; peptide antibiotic; phenicol antibiotic;s ulfonamide antibiotic; tetracycline antibiotic	Antibiotic efflux
**Upregulated ARGs**							
***dapL***	LL-diaminopimelate aminotransferase	2.006	E	ROSA	Peptide	Peptide antibiotic	Antibiotic efflux
***yecD***	Isochorismatase family protein YecD	1.1223	Q	OMPR	Beta-lactam	Carbapenem; penam; cephalosporin; penem; cephamycin; monobactam	Reduced permeability to antibiotics
***pse_5134***	Cro Cl family transcriptional regulator	1.212	K	MEXL	Multidrug	Macrolide antibiotic; tetracycline antibiotic; triclosan	Antibiotic efflux
***dctD_4***	C4-dicarboxylate transport transcriptional regulator	1.1834	T	RPSD_(RAMA_OR_SUD2)	Unclassified	Unclassified	-

Genes/transcripts least expressed (with log_2_ fold change below −0.5 and *p*-value lower than 0.05) under TH adaptation (**negative**). Genes/transcripts most expressed (with log_2_ fold change over 0.5 and *p*-value lower than 0.05) under TH adaptation (**positive**).

**Table 4:** Differentially detected metabolites by GC-ToF-MS in TH-adapted *Pseudomonas psychrophila* M33T02.2.

Metabolite	log_2_ FC	*p*-Value	−log_10_ *p*-Value
Maltose	−0.592	0.0019	2.717
Histidine	0.718	0.0402	1.395
GABA	−0.559	0.0100	2.002
Malic acid	−1.098	0.0102	1.990
Fumaric acid	−0.532	0.0225	1.648

## Data Availability

The original contributions presented in this study are included in the article/Supplementary Material. Further inquiries can be directed to the corresponding author.

## Supplementary Materials

The following supporting information can be downloaded at: https://www.mdpi.com/article/10.3390/ijms27093777/s1. References [53,54,55,56,57] are cited in the Supplementary Materials.
