# Supplementary figures and images for: Integrated Transcriptomics and Metabolomic Profiling Suggests That Thymol Adaptation Induces Multi-Layered Envelope and Metabolic Perturbations That Sensitize Pseudomonas psychrophila to Antibiotics
Source: Int J Mol Sci. 2026 Apr 23;27(9):3777. doi: 10.3390/ijms27093777 (PMC13163456; doi:10.3390/ijms27093777)

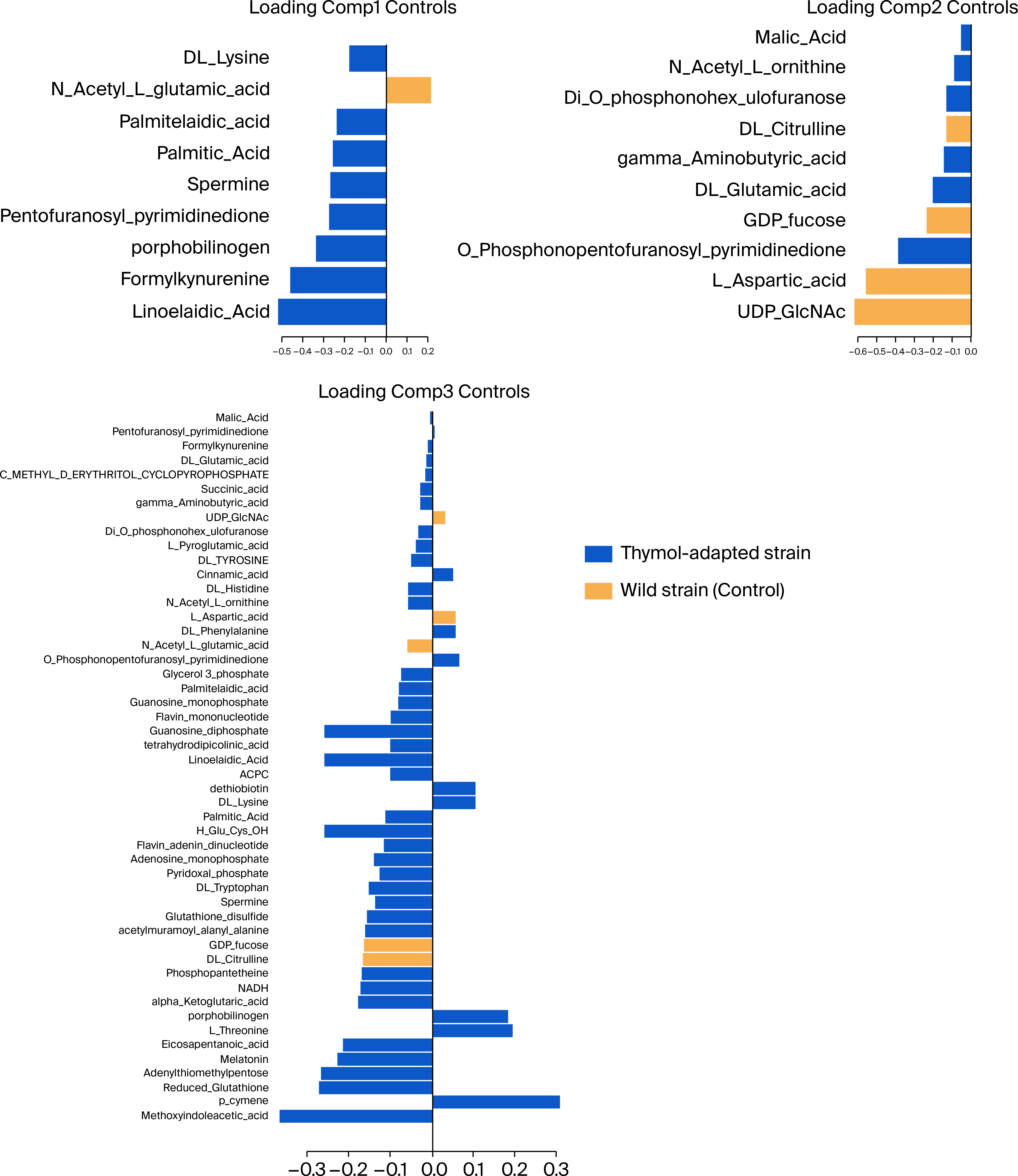

Supplement: Supplementary file 1 [file ijms-27-03777-s001.zip › Figure-S1.png]
